# *In Silico* Screening and Validation of PDGFRA Inhibitors Enhancing Radioiodine Sensitivity in Thyroid Cancer

**DOI:** 10.3389/fphar.2022.883581

**Published:** 2022-05-12

**Authors:** Xuefei Yu, Xuhang Zhu, Lizhuo Zhang, Jiang-Jiang Qin, Chunlai Feng, Qinglin Li

**Affiliations:** ^1^ School of pharmacy, Jiangsu University, Zhenjiang, China; ^2^ The Cancer Hospital of the University of Chinese Academy of Sciences (Zhejiang Cancer Hospital), Institute of Basic Medicine and Cancer (IBMC), Chinese Academy of Sciences, Key Laboratory of Head & Neck Cancer Translational Research of Zhejiang Province, Hangzhou, China; ^3^ Institute of Basic Medicine and Cancer (IBMC), Chinese Academy of Sciences, Hangzhou, China; ^4^ Thyroid surgery, The Cancer Hospital of the University of Chinese Academy of Sciences (Zhejiang Cancer Hospital), Institute of Basic Medicine and Cancer (IBMC), Chinese Academy of Sciences, Hangzhou, China; ^5^ Department of Head and Neck Surgery, Center of Otolaryngology-Head and Neck Surgery, Zhejiang Provincial People’s Hospital (People’s Hospital of Hangzhou Medical College), Hangzhou, China

**Keywords:** PDGFRA inhibitors, virtual screening, radioiodine-refractory thyroid cancer, traditional Chinese medicine, machine learning

## Abstract

Aberrant activation of platelet-derived growth factor receptor α (PDGFRA) has been implicated in tumorigenesis and radioiodine resistance of thyroid cancer, indicating its therapeutic potential. In the present study, we confirmed the association between PDGFRA and radioiodine resistance in thyroid cancer using bioinformatics analysis and constructed a prediction model of PDGFRA inhibitors using machine learning and molecular docking approaches. We then performed a virtual screening of a traditional Chinese medicine (TCM) derived compound library and successfully identified 4’,5,7-trimethoxyflavone as a potential PDGFRA inhibitor. Further characterization revealed a significant inhibitory effect of 4’,5,7-trimethoxyflavone on PDGFRA-MAPK pathway activation, and that it could upregulate expression of sodium iodide symporter (NIS) as well as improve radioiodine uptake capacity of radioiodine-refractory thyroid cancer (RAIR-TC), suggesting it a potential drug lead for the development of new RAIR-TC therapy.

## 1 Introduction

Thyroid cancer is one of the most common endocrine system malignancies, especially in women. The latest global malignancies statistics from the International Agency for Research on Cancer (GLOBOCAN 2020) revealed thyroid cancer the 9th most common malignant tumor in the world and the 5th most common among women. With an estimated 586,000 new cases and 44,000 deaths in 2020, the incidence of thyroid cancer has been on the rise (Bray et al., 2018; Miller et al., 2020; Sung et al., 2021). Differentiated thyroid cancer (DTC) is the most common thyroid cancer. As it retains the function of thyroid follicular cells to a certain extent, most DTC patients are treated in standard surgical and radioactive iodine (RAI) treatments with good prognosis, especially when DTC is limited to the thyroid or only involves the cervical lymph nodes (Ferlay et al., 2019). However, the risk of local recurrence and distant metastasis could be as high as 20 and 10%, which has been the main cause of death in thyroid cancer patients (Anderson et al., 2013; Liu et al., 2019). The radioiodine uptake characteristics of patients with metastatic lesions are positively correlated with their treatment prognosis (van Tol et al., 2003). As statistics showed, the 10-year survival rate of patients with aberrant radioiodine uptake has been much lower than that of patients with radioiodine uptake (Durante et al., 2006). Therefore, improving the radioiodine uptake of RAIR-TC has been considered one of the most effective treatments.

PDGFRA is a cell surface receptor tyrosine kinase. PDGFRA can bind to its corresponding ligand PDGF and then activate downstream signaling pathways to regulate cell proliferation, migration and angiogenesis (Heldin and Westermark, 1999; Heldin and Lennartsson, 2013; Roskoski, 2018). The overexpression of PDGFRA is closely associated with radioiodine resistance (Lopez-Campistrous et al., 2016) and distant metastasis (Lin et al., 2021) in human thyroid cancer. Lopez-campistou et al. have (Lopez-Campistrous et al., 2016) found that overexpression and/or aberrant activation of PDGFRA decreased the expression levels of TG and NIS by disrupting the transcriptional activity and nuclear localization of TTF1 in both cell and animal models. When PDGFRA was inhibited, the uptake of radioiodine was restored, and the migration, invasion potential and tumor burden were also reduced. Currently, sorafenib (Brose et al., 2014), which can target PDGFRA, has been approved for marketing by National Medical Products Administration, while Lenvatinib (Schlumberger et al., 2015; Porcelli et al., 2021) and Pazopanib (Chow et al., 2017) have entered the stage of clinical research. However, these medications are multi-targeted tyrosine kinase inhibitors, and further development of selective PDGFRA inhibitors is in urgent need.

Over the years, TCM has achieved certain clinical success in the treatment of thyroid cancer. According to the different symptoms of thyroid cancer before and after the operation, the corresponding TCM treatment can significantly improve the patients’ quality of life (Chang and Li, 2018; Han et al., 2021). At the same time, TCM can also improve the uptake rate of radioiodine in thyroid cancer cells. Mechanism studies have revealed an important role of TCM in regulating NIS expression and improving radioiodine uptake (Yu et al., 2013; Hardin et al., 2016). In this study, we aimed to identify new PDGFRA inhibitors from TCM *in silico* and verify their effects of targeting PDGFRA and radioiodine uptake.

## 2 Materials and Methods

### 2.1 Correlation Between RDGFRA and Radioiodine Uptake of Thyroid Cancer

#### 2.1.1 Expression Analysis of PDGFRA in Oncomine Database

To investigate the expression difference of *PDGFRA* in RAIR-TC and radioiodine-sensitive thyroid cancer, the expression of *PDGFRA* in different thyroid cancer was collected by inputting two keywords *PDGFRA* and thyroid cancer in the Oncomine database (https://www.oncomine.org/). Since DTC was not subdivided into radioiodine-sensitive and radioiodine-refractory type in the public database, DTC was regarded as radioiodine-sensitive type. GraphPad Prism version 8 (GraphPad Software, California, United States) was used for mapping.

#### 2.1.2 Protein-Protein Interaction (PPI) Networks Analysis

Protein-protein interaction (PPI) networks functional enrichment analysis was executed using the searching tool for recurring instances of neighbouring genes (STRING) 11.0 (https://string-db.org/), which is used to explore interactions among proteins. The protein names or sequences of PDGFRA were input, and the sample type of “homo sapiens” was selected. The results of PPI networks were downloaded.

#### 2.1.3 Overall Survival Analysis of *PDGFRA*

To investigate relationship between *PDGFRA* and prognosis of thyroid cancer, the overall survival analysis of *PDGFRA* was conducted. The original data for survival analysis were obtained from the cBioPortal database (https://www.cbioportal.org/). The expression value of *PDGFRA* in the top 25% was considered as high expression, and the rest was intermediate or low expression. Then, the overall survival-time plot of *PDGFRA* was assessed by Kaplan-Meier plot and Log-Rank analysis.

#### 2.1.4 Correlation Analysis Between *PDGFRA* and Thyroid-specific Genes

To further investigate the correlation between *PDGFRA* and radioiodine uptake capacity of thyroid cancer, correlation analysis between *PDGFRA* and thyroid-specific genes (*NIS*, *PAX8*, *TTF1*) were performed using R commands. The expression data of *PDGFRA*, *NIS*, *PAX8*, and *TTF1* were obtained from cBioPortal database. Pearson correlation coefficient was used to evaluate the correlation between thyroid-specific genes and *PDGFRA*.

### 2.2 Screening of Novel Inhibitors of PDGFRA

**FIGURE 1:**
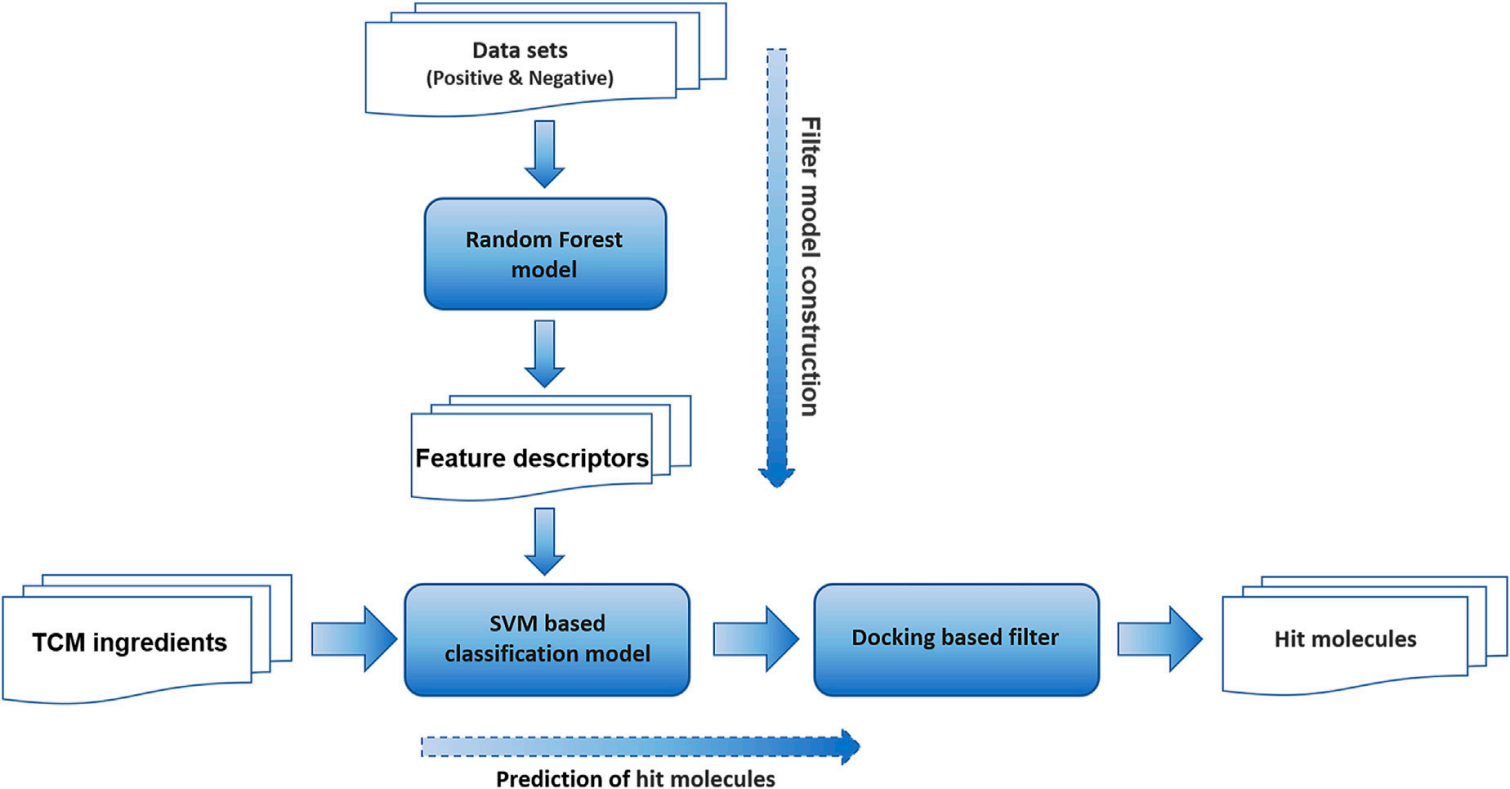
The virtual screening workflow adopted in this study.

The virtual screening workflow adopted in this study is shown in Figure 1.

#### 2.2.1 Construction of the Data Sets

All the 504 PDGFRA inhibitors were collected from the Binding Database (http://www.bindingdb.org/bind/index.jsp). Among them, 423 compounds with IC_50_ ≤10 μM were considered active and the rest were considered inactive. Another inactive dataset of 496 molecules was collected from the CHEMBL database (https://www.ebi.ac.uk/chembl/), which was randomly selected as an assumed non-inhibitor dataset. Subsequently, a set of 423 compounds as active molecules were taken, and the remaining 81 inactive molecules along with 496 randomly selected molecules constituted the inactive dataset. Both active dataset and inactive dataset were randomly divided into training and test set in a 4:1 ratio. Thus, we have 800 compounds in the training set, out of which 345 were active ones, the remaining 455 were inactive. Likewise, we have a total of 200 compounds in the test set, out of which 78 were active and the remaining 122 were inactive (Table 1).

**TABLE 1:** Compounds for model construction.

Category	Label		
	Positive	Negative	Total number
Training set	345	455	800
Test set	78	122	200
Screening set	—	—	2994

A total of 2994 Chinese herbal ingredients from 116 Chinese herbal medicines used in thyroid cancer treatment and patient physical quality regulation were collected from Traditional Chinese Medicine Systems Pharmacology Database and Analysis Platform (TCMSP, https://old.tcmsp-e.com/tcmsp.php) and Traditional Chinese Medicine Integrated Database (TCMID, http://www.megabionet.org/tcmid/) as a screening set (Table 1).

#### 2.2.2 Random Forest Model-Based Feature Screening

To obtain the feature descriptors of compounds, Random Forest model was constructed. First, a total of 354 descriptors for the compounds were calculated using the Molecular Operating Environment 2020 (MOE, Chemical Computing Group ULC, Montreal, Canada) after energy minimization (Vilar et al., 2008). Then, the training set was used to train the Random Forest model, and the parameters “n_estimator”, “max_depth”, “min_sample_split”, and “min_sample_leaf” were optimized to determine the best parameters of the Random Forest model. Finally, The Random Forest model was constructed by using the best parameters, and the model performance was investigated by the test set. Descriptors with the feature importance scores greater than 0.0037 calculated by the model were selected as feature descriptors.

#### 2.2.3 Support Vector Machine (SVM) Based Prediction Model

Based on the feature descriptors screened by Random Forest model, SVM based prediction model was constructed with radial basis function (RBF) kernel function. Two hyperparameters (c, γ) were optimized by grid search and 7-fold cross-validation (7-CV). After generating the SVM based prediction model using 7-CV of the training set, the generated model was further validated using the test set. The statistical parameters Accuracy (ACC), Precise (P), and Recall (R) were calculated to test the validity of generated model.

#### 2.2.4 Molecular Docking

All the molecular docking studies were carried out by MOE. The crystal structure of PDGFRA (PDB ID: 5K5X) was selected for the docking studies and retrieved from the RCSB Protein Data Bank (PDB, https://www.rcsb.org/; Bahmani et al., 2021). The 3D structure of screened compounds was retrieved from the PubChem database (https://pubchem.ncbi.nlm.nih.gov/). After the protein and compounds were pretreated separately, the binding sites on the receptor and compounds were studied by blind docking. Finally, for all compounds, the lowest energy conformation was used for further analysis.

### 2.3 *In vitro* Biological Activity Evaluation of Screened PDGFRA Inhibitors

#### 2.3.1 Cell Culture

Cell lines Nthy-ori-3-1, BCPAP, TPC1, 8505C, and IHH4 were obtained from Zhejiang Provincial People’s Hospital (Hangzhou, China). Nthy-ori-3-1, BCPAP, TPC1, and IHH4 cell lines were maintained in RPMI 1640 supplemented with 10% FBS plus 1% penicillin-streptomycin solution, while 8505C cells were maintained in DMEM supplemented with 10% FBS plus 1% penicillin-streptomycin solution. All the cell lines were cultured at 37°C in a humidified 5% CO_2_ incubator.

#### 2.3.2 Cell Viability Assay

Cells were incubated in 96-well plates at a density of 5×10^3^ cells/well for 24 h. The cells were treated with the indicated concentrations of compounds for 72 h and then incubated with Cell Counting Kit-8 (CCK-8) reagent (Biosharp, Anhui, China) at 37°C for 2 h. The absorbance was measured at 450 nm on the Spark^®^ multimode microplate reader (Tecan, Männedorf, Switzerland). All compounds were purchased from Shanghai Red Peony Biotechnology Co., LTD. (Shanghai, China).

#### 2.3.3 Western Blot Analyses

The treated IHH4 cells were lysed with a cell lysate containing RIPA buffer [50 mM Tris (pH 7.4), 150 mM NaCl, 1% Triton X-100, 1% sodium deoxycholate, 0.1% Sodium dodecyl sulfate (SDS), sodium orthovanadate, sodium fluoride, Ethylene Diamine Tetraacetic Acid (EDTA), leupeptin et al.], supplemented with 1 mM phenylmethylsulphonyl fluoride (PMSF) and 10% phosphatase inhibitors on ice for 15 min. The protein extract was obtained after centrifugation at 4°C at 13,000 rpm for 15 min. The protein concentration was evaluated using Pierce BCA protein assay reagent (Biosharp, Anhui, China). Running samples were prepared by adding a sample diluent and 5× SDS-polyacrylamide gel electrophoresis (SDS-PAGE) loading buffer, and the protein denaturation is performed at 100°C for 10 min. The protein extract samples were separated by SDS-PAGE and transferred to 0.45 μm polyvinylidene difluoride (PVDF) membrane. Then, membranes were blocked with 5% skimmed milk powder in 1× Tris-Buffered Saline and Tween 20 (TBST) for 1 h, followed by incubation with primary antibodies at 4°C overnight. After being cleaned 3 times with TBST, the protein bands were incubated with anti-mouse or anti-rabbit IgG horseradish peroxidase (HRP) linked secondary antibody (Cell Signaling Technology, Danvers, United States) for 2 h. After being washed 3 times with TBST, the protein bands were finally visualized with SuperSignal West Pico chemiluminescence substrate (Biosharp, Anhui, China). The antibodies used for the western blot were as follows: monoclonal antibodies against phospho-PDGFRA (Tyr754, Cat. No. 2992), Na^+^/K^+^ ATPase (Cat. No. 3010), and GAPDH (Cat. No. 5174) were purchased from Cell Signaling Technology (Danvers, United States). The PDGFRA (Cat. No. ab134123) and NIS (Cat. No. ab242007) monoclonal antibody was purchased from Abcam (Cambridge, United States). The p38 MAPK (Cat. No. sc-7972) and phospho-p38 MAPK (Tyr 182, Cat. No. sc-7973) monoclonal antibodies were purchased from Santa Cruz Biotechnology (Dallas, United States). All antibodies were diluted at 1:1000 in the study.

#### 2.3.4 Cellular Thermal Shift Assay (CETSA)

CETSA is used to detect the binding of compounds to PDGFRA (Martinez Molina et al., 2013). Cells were incubated in 15 cm cell culture plates at a density of 3×10^7^ cells/well for 24 h. The cells were treated with the indicated concentrations (0, 80 μM) of compounds for 1.5 h and then cells were digested with trypsin and collected by centrifugation. After washing with PBS, the cells were re-suspended with 1 ml PBS containing 1% PMSF. The cell suspension was placed in a series of PCR tubes (control vs. treated) and then subjected to thermal shock for 3.5 min in an appropriate thermal cycle (37–67°C), followed by incubation at room temperature for 3min. The suspensions were then subjected to two freeze-thaw cycles in liquid nitrogen, and vortex treatment was carried out after each thawing. The suspensions were centrifuged at 4°C for 20 min at 2,000 rpm and the supernatants were transferred to new PCR tubes. Running samples were prepared by adding SDS-PAGE loading buffer and then heated to denature at 100°C for 10 min. Finally, the proteins were analyzed by western blot.

#### 2.3.5 Radioiodine Uptake

Radioiodine uptake experiments at the cellular level were performed as reported by Weiss (Weiss et al., 1984) with appropriate adjustments. 1×10^6^ cells were cultured in 6-well plates and treated with compounds for 24 h after adherence. Then, the cells were washed with phosphate-buffered saline (PBS) 3 times. One group of experimental cells was cultured with serum-free medium at 37 °C for 2 h, digested by trypsin, and counted by hemocytometer. The other group of parallel cells was added 1 ml serum-free medium containing 1 μCi Na^131^I and cultured at 37°C for 2 h. The cells were washed by precooled PBS 3 times, digested by trypsin, and the cell suspension was collected by releasing free tubes. Counts per minute (CPM) of radioactivity was measured using GC-1500 γ radiation immunity arithmometer (ZONKIA, Anhui, China) and the effect of compounds on radioiodine uptake was evaluated using CPM/10^5^ cells as the unit of cellular radioiodine uptake.

### 2.4 Statistical Analysis

All statistical analysis were done using GraphPad Prism version 8. Survival Analysis were performed using Statistical Product and Service Solutions 20 (SPSS, IBM, United States) software. Significance of the difference was calculated by independent-Sample t-test. A *p*-value <0.05 was considered statistically significant. All data in the figures are presented as means ± SD.

## 3 Results

### 3.1 Correlation Between PDGFRA and Radioiodine Uptake of Thyroid Cancer

**FIGURE 2:**
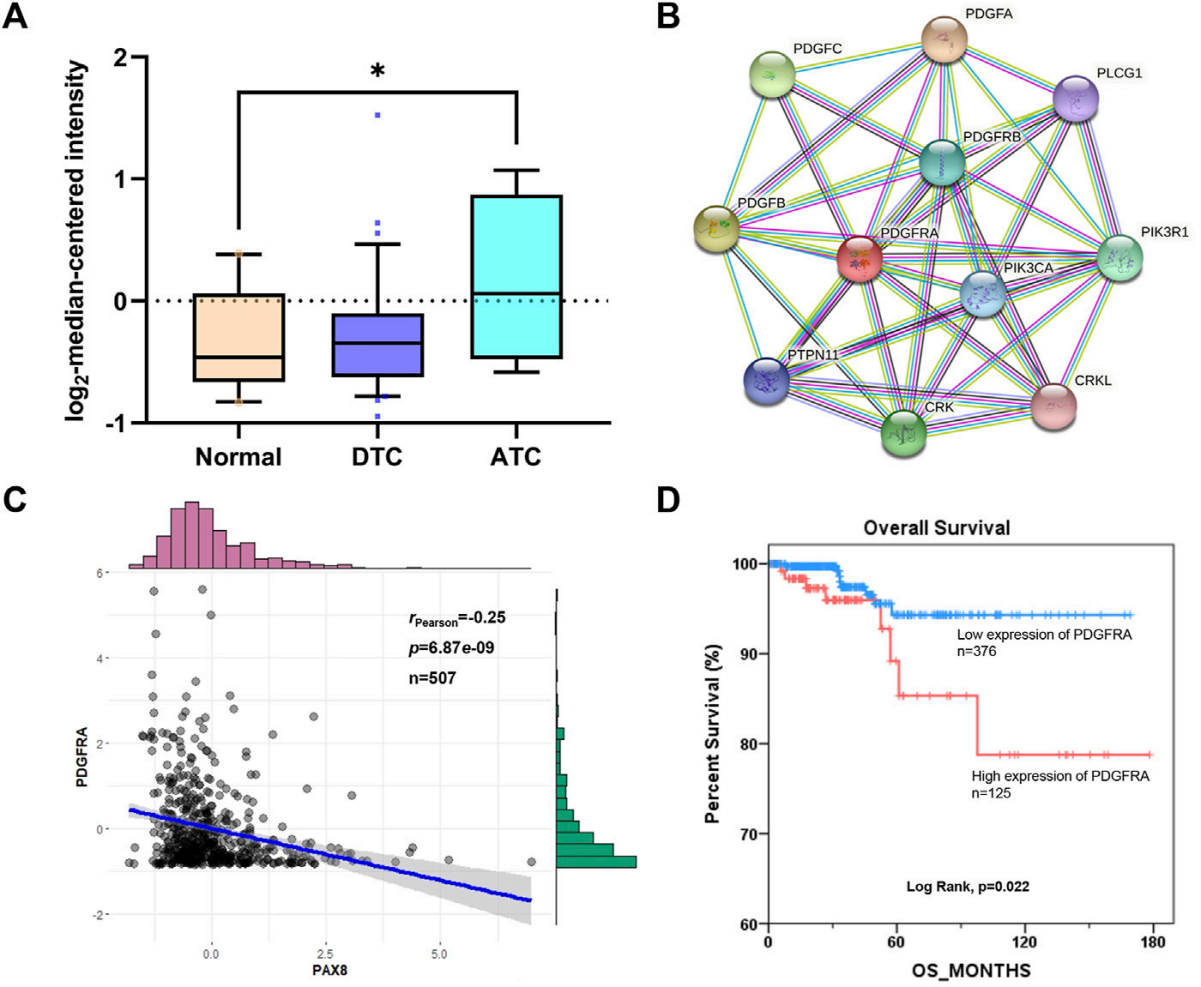
The correlation between RDGFRA and radioiodine uptake of thyroid cancer. **(A)** The expression of *PDGFRA* in different types of thyroid cancer from Oncomine database. *, *p* <0.05, compared with normal group. **(B)** The biological correlation between PDGFRA and related regulatory genes was analyzed based on STRING database. **(C)** Correlation between *PDGFRA* and *PAX8* based on cBioPortal database. **(D)** Kaplan-Meier plot of overall survival-time of *PDGFRA* mRNA expression based on cBioPortal database. DTC: Differentiated Thyroid Cancer; ATC: Anaplastic Thyroid Cancer.

To confirm the correlation between RDGFRA and radioiodine uptake of thyroid cancer, the expression of *PDGFRA*, protein-protein interaction network, correlation with thyroid-specific genes, and survival differences were analyzed. As shown in Figure 2A, anaplastic thyroid carcinoma (ATC) is a type of RAIR-TC due to poor radioiodine uptake. The expression of *PDGFRA* in ATC is significantly higher than that in normal thyroid cells, which confirmed that the expression of *PDGFRA* in RAIR-TC is higher than that in normal thyroid or radioiodine-sensitive thyroid cancer. PPI networks (Figure 2B) showed a strong interaction between PDGFRA and PI3K (Score >0.9). PI3K pathway has been confirmed to affect radioiodine uptake of thyroid cells (Kogai et al., 2008; Liu et al., 2012), which is another evidence that PDGFRA is related to the uptake of radioiodine. Correlation analysis between *PDGFRA* and thyroid-specific genes showed that although the expression of *PDGFRA* was not correlated with *NIS* and *TTF1*, it was negatively correlated with *PAX8* (Figure 2C, Supplementary Figure S1). Kaplan-Meier plot of overall survival-time of *PDGFRA* mRNA expression showed that high expression of *PDGFRA* affected the prognosis of patients (Figure 2D). In summary, PDGFRA plays an important role in the uptake or transport of radioiodine in thyroid cancer and is a reliable target for reversing RAIR-TC radioiodine resistance.

### 3.2 Selection of Feature Descriptors Based on Random Forest Model

**FIGURE 3:**
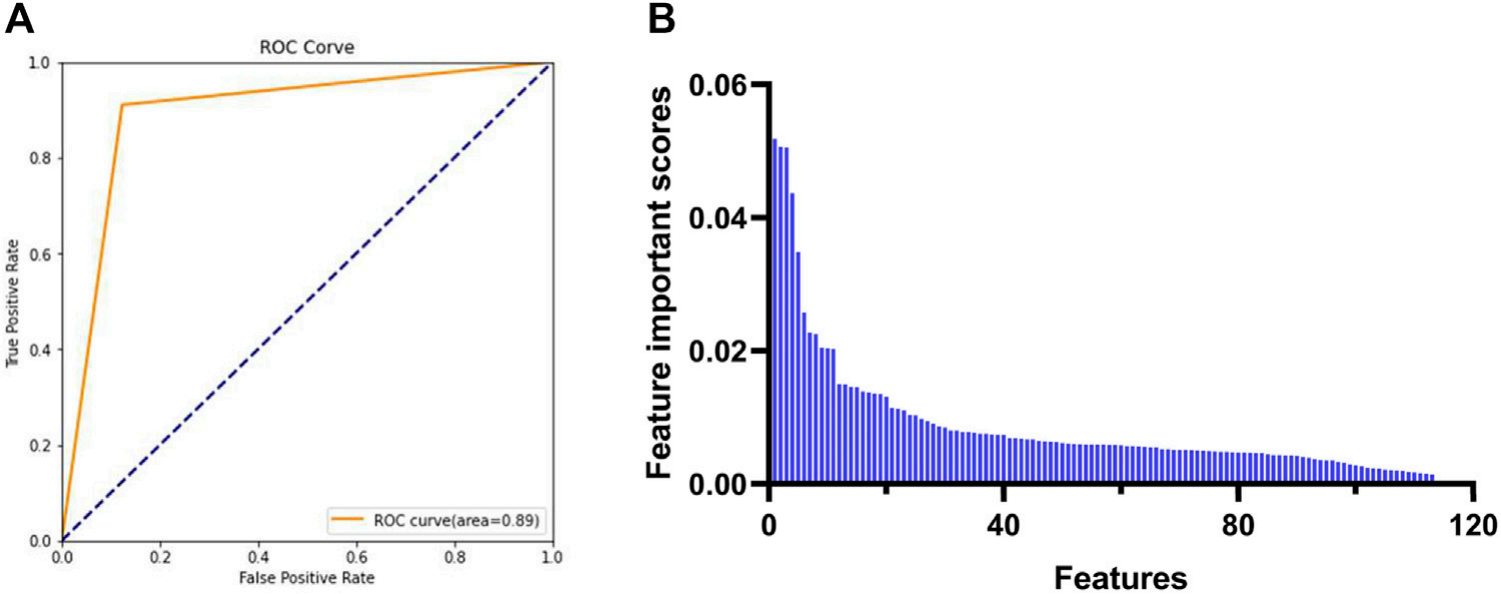
Selection of feature descriptors based on Random Forest model. **(A)** AUC of Random Forest model under the best parameters. **(B)** Feature importance scores calculated by the Random Forest model. AUC: area under receiver operating characteristic curve.

To obtain the feature descriptors, the molecular descriptors of a preconstructed dataset consisting of 423 known PDGFRA inhibitors (positive) and 577 non-inhibitors (negative) were calculated by MOE software, as shown in Supplementary Tables S1, S2. In order to increase the accuracy of the subsequent model and reduce the amount of computation, the initial 354 descriptors were preprocessed. Descriptors with null or missing values, a repeat rate of more than 80% and a standard deviation of less than or equal to 0.05, and highly correlated with others (correlation coefficients >80%) were all removed. Finally, 117 molecular descriptors were chosen for building the Random Forest model. The Grid Search result showed that when the values of parameters n_estimator, max_depth, min_sample_split, and min_sample_leaf were 177, 17, 2, and 1, respectively, the model demonstrated the best accuracy (89%) and AUC (area under receiver operating characteristic curve, 0.89) (Table 2 and Figure 3A). Finally, 93 feature descriptors were selected based on the feature importance scores calculated by the Random Forest model, which were shown in Table 3 and Figure 3B.

**TABLE 2:** Optimal parameters and overall Performance of Random Forest Prediction Model.

v	n_estimator	max_depth	min_sample_split	min_sample_leaf	ACC (%)	P	R (%)
7	177	17	2	1	89	83%	91

v: Number of folds for cross-validation; ACC: accuracy; P: precision; R: recall.

**TABLE 3:** The 93 molecular descriptors filtered by the Random Forest model.

Descriptor class	Descriptors	Number
Physical properties	apol, h_logD, rsynth	3
Subdivided surface areas	SlogP_VSA0,SlogP_VSA1,SlogP_VSA2,SlogP_VSA3,SlogP_VSA4,SlogP_VSA5,SlogP_VSA9,SMR_VSA1,SMR_VSA2,SMR_VSA3,SMR_VSA4,SMR_VSA7	12
Atom counts and bond counts	a_aro,a_ICM,a_nN,a_nS,b_1rotN,b_1rotR,b_max1len,chiral,opr_brigid	9
Partial charge descriptors	PEOE_RPC+,PEOE_RPC-,PEOE_VSA+0,PEOE_VSA+1,PEOE_VSA+2,PEOE_VSA+3, PEOE_VSA-0,PEOE_VSA-1,PEOE_VSA-3,PEOE_VSA-4,PEOE_VSA-5,PEOE_VSA-6, PEOE_VSA_FHYD, PEOE_VSA_FNEG,Q_RPC+,Q_RPC-	16
Pharmacophore feature descriptors	a_don, vsa_acc, vsa_don, vsa_other	4
Adjacency and distance matrix descriptors	BalabanJ,BCUT_PEOE_0,BCUT_SLOGP_1,GCUT_PEOE_1,GCUT_SLOGP_0,GCUT_SLOGP_1	6
Potential energy descriptors	E,E_ang,E_ele,E_oop,E_sol,E_tor	6
MOPAC descriptors	MNDO_dipole	1
Surface area descriptors	dens,glob,npr1,pmi1,pmiX,pmiY,pmiZ,std_dim2,std_dim3,vsurf_A,vsurf_CP,vsurf_CW2,vsurf_EDmin1,vsurf_EWmin1,vsurf_HB1,vsurf_HL1,vsurf_IW1,vsurf_IW7,vsurf_IW8	19
Conformation dependent Charge Descriptors	ASA+,ASA-,ASA_P,CASA-,dipole,dipoleX,dipoleY,dipoleZ,FASA+,FASA-	10
Hueckel theory descriptors	h_ema,h_logD,h_pavgQ,h_pKa,h_pKb,h_pstates,h_pstrain	7

### 3.3 Construction, Evaluation of SVM Based Prediction Model, and Prediction of PDGFRA Potential Inhibitors

**FIGURE 4:**
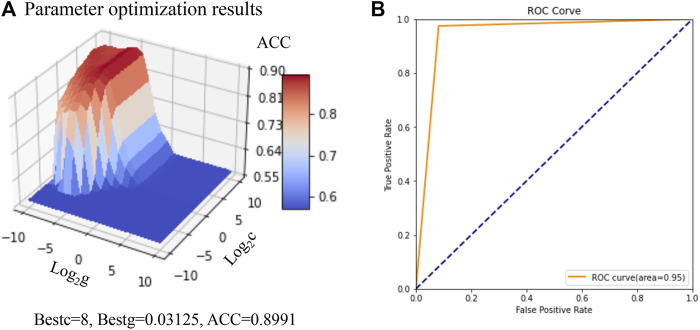
Construction and optimization of SVM based prediction model. **(A)** Parameter optimization results based on Grid search and cross validation. **(B)** AUC of SVM based prediction model under the best parameters. AUC: area under receiver operating characteristic curve.

Based on 93 feature descriptors selected by the Random Forest model constructed above, the initial SVM based prediction model was constructed. The parameters of the SVM based prediction model were optimized based on the grid search algorithm and 7-fold cross validation, and the ACC, P, R and AUC of the model was taken as the evaluation index. When the value of parameters c was 8 and the value of parameters γ was 0.03135, the model demonstrated the best accuracy (90%) for the training set (Figure 4A). When the optimized model was used for the prediction of the test set, the prediction accuracy was 94%, while the AUC was 0.95 (Table 4 and Figure 4B). 97% of the positive samples were properly predicted for the 78 PDGFRA inhibitors, while for the 122 non-inhibitors, 92% of the negative samples were properly predicted. Collectively, these results confirmed that the constructed SVM classification model had a greatly good capability to distinguish the PDGFRA inhibitors and non-inhibitors.

**TABLE 4:** Optimal parameters and overall Performance of SVM based prediction model.

v	c	Gamma	Kernel	ACC (%)	P (%)	R (%)
7	8	0.03135	rbf	94	88	97

v: Number of folds for cross-validation; ACC: accuracy; P: precision; R: recall.

The optimal SVM based prediction model was then used to screen potential PDGFRA inhibitors. 30 of 2994 compounds passed through this filter were predicted as potential PDGFRA inhibitors, which were shown in Table 5.

**TABLE 5:** Screening results of active TCM ingredients based on SVM based prediction model.

CID	Name	TCM name	CID	Name	TCM name
11066	Oxyberberine	Coptidis Rhizoma	5281636	Gentisin	Dipsaci Radix
68077	Tangeretin	Citrus Reticulata、Citri Reticulatae Pericarpium Viride, Aurantii Fructus Immaturus	5281704	Afrormosin	licorice、Spatholobus Suberectus Dunn
79730	4',5,7-Trimethoxyflavone	Aurantii Fructus Immaturus	5317756	Glycycoumarin	licorice
124050	Isoglycyrol	Licorice	5319013	Licoricone	licorice
150032	Menisporphine	Phellodendri Chinrnsis Cortex	5319422	3'-Methoxydaidzein	Polygonati Rhizoma
160921	Nevadensin	Asparagi Radix	5319744	3'-O-Methylorobol	Ecliptae Herba
161271	Salvigenin	Scutellariae Barbatae Herba、Scutellariae Radix	5320083	Glycyrol	licorice、Amygdalus Communis Vas
161748	Myricanone	Chuanxiong Rhizoma	5320290	Onjixanthone I	Forsythiae Fructus
185034	Sainfuran	Radix Bupleuri	5352005	Retusin	Agastacherugosus (Fisch.etMey)O.Ktze
442694	Batatasin I	Rhizoma Dioscoreae	11983285	Confusarin	Dendrobium nobile Lindl
480787	Glycyrin	licorice	13965473	Odoratin	licorice、Spatholobus Suberectus Dunn
480817	Gancaonin V	licorice	13970974	4,6-Dimethoxy-7-(3-methylbut-2-enoxy)furo [2,3-b]quinoline	Dendrobium nobile Lindl
629964	4',5,7,8-Tetramethoxyflavone	Aurantii Fructus Immaturus	14187587	Isoglycycoumarin	licorice
688717	3-Hydroxy-2',4',7-trimethoxyflavone	Lonicerae Japonicae Flos	14353376	5-Hydroxy-7,8,4'-trimethoxyflavone	Scutellariae Barbatae Herba
5281601	Apigenin dimethylether	Scutellariae Barbatae Herba、Lonicerae Japonicae Flos、Epimrdii Herba	44257530	Phaseol	licorice、Amygdalus Communis Vas

### 3.4 Molecular Docking Study

**FIGURE 5:**
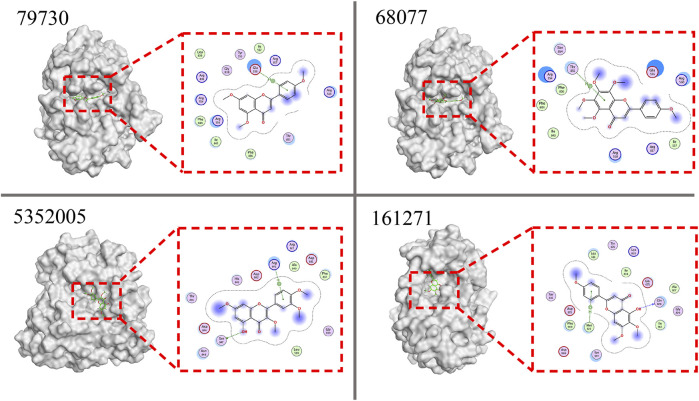
Docking results of the top 4 compounds with PDGFRA. 79730, 68077, 5352005, and 161271 are PubChem CID of 4’,5,7-Trimethoxyflavone, Tangeretin, Retusin, and Salvigenin, respectively. PDGFRA protein is shown as a white surface model, while the ligand is shown as a green stick model. The hydrogen bond is shown as the dotted lines. In the 2D interaction diagram, the ligand is shown as a chemical formula, the residues are shown in purple circles marked with their names, and the hydrogen bond is shown as the green dotted lines.

To evaluate the interactions of 30 candidate compounds screened based on the SVM based prediction model with PDGFRA, molecular docking study was carried out by MOE software. The active site of PDGFRA mainly consists of three parts, including ATP binding site, activation loop, and the amino acids available active site (Bahmani et al., 2021). Compounds that bind to the active sites of PDGFRA may be more effective in inhibiting PDGFRA. The docking results of compounds and PDGFRA were shown in Table 6. Nineteen compounds were hydrogen-bonded to the reported PDGFRA active region. Among the nineteen compounds, the top 10 compounds available for purchase were selected for subsequent experimental verification according to the principle of the lowest scores (Figure 5, Supplementary Figure S2).

**TABLE 6:** Docking results of potential inhibitors with PDGFRA.

CID	S	NHB^a^	Binding site	CID	S	NHB^a^	Binding site
11066	−6.03787	1	GLU556	5281636	−5.09768	2	ILE965/SER783
68077	−6.36916	1	THR855	5281704	−5.76621	1	ARG560
79730	−6.50424	1	GLU556	5317756	−6.18588	0	—
124050	−5.93374	0	—	5319013	−6.12188	0	—
150032	−5.51394	0	—	5319422	−5.3683	2	GLU556/ARG554
160921	−5.67415	0	—	5319744	−5.35448	0	—
161271	−6.12935	2	GLN828/MET622	5320083	−5.89688	1	ARG554
161748	−6.14682	1	LYS833	5320290	−5.55545	0	—
185034	−5.70737	0	—	5352005	−6.20545	2	SER847/ARG841
442694	−5.52984	1	ARG560	11983285	−5.63999	1	ARG817
480787	−5.8909	2	GLU556/ARG817	13965473	−5.63319	0	—
480817	−5.84344	1	ASP968	13970974	−5.84314	2	ARG554/THR855
629964	−6.08919	2	TYR679/GLY680	14187587	−6.21415	0	—
688717	−6.19365	1	ARG597	14353376	−5.47657	2	GLU675/ILE965
5281601	−5.77296	1	ARG560	44257530	−5.74329	0	—

a NHB: number of hydrogen bonds.

### 3.5 *In vitro* Activity Evaluation of Screened PDGFRA Inhibitors

**FIGURE 6:**
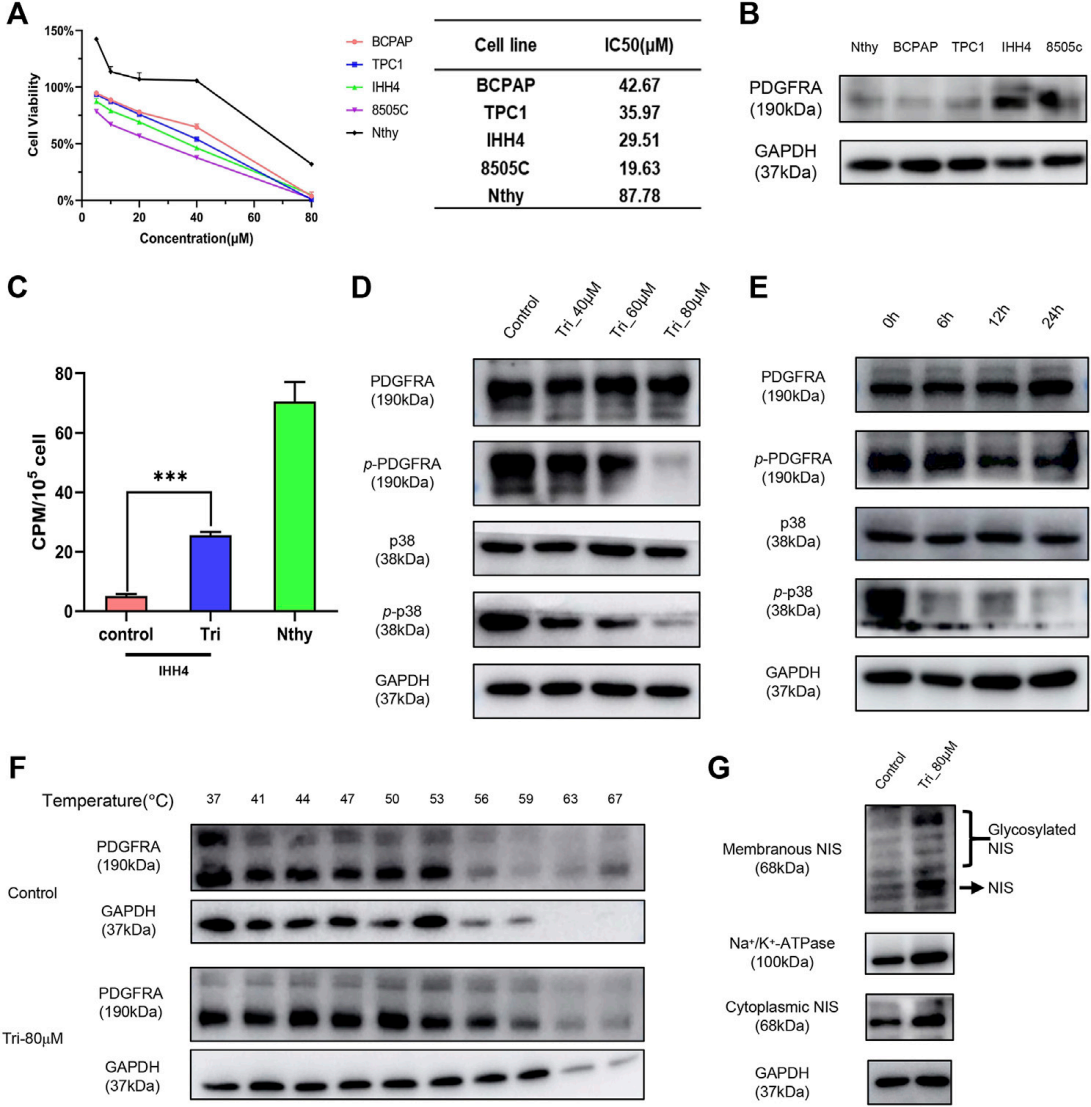
Biological validation of the virtually screened PDGFRA inhibitors. **(A)** The effect of 4’,5,7-trimethoxyflavone on the viability of thyroid carcinoma cells and normal thyroid cells. **(B)** The expression of PDGFRA in thyroid carcinoma cells and normal thyroid cells. **(C)** The effect of 4’,5,7-trimethoxyflavone on radioiodine uptake capacity of IHH4 cell line. **(D,E)** The effect of 4’,5,7-trimethoxyflavone on protein expression of p-PDGFRA and p-p38. **(F)** The binding of 4’,5,7-trimethoxyflavone to PDGFRA was examined by the CETSA test. **(G)** The effect of 4’,5,7-trimethoxyflavone on expression and cellular location of NIS. ***, *p* <0.001, compared with control group. Nthy: Nthy-ori-3-1 cell line; Tri: 4’,5,7-trimethoxyflavone; p-PDGFRA: phospho-PDGFRA (Tyr754); p-p38: phospho-p38 MAPK (Tyr 182).

To confirm that the previously screened inhibitors can inhibit PDGFRA and improve the radioiodine uptake capacity of thyroid cancer, biological validation of the selected inhibitors was carried out. In this study, normal thyroid epithelial cell line Nthy-ori-3-1, DTC cell lines BCPAP, TPC1, and IHH4, and ATC cell line 8505C were used as cell models. The effects of ten potential PDGFRA inhibitors on the viability of these cell lines were fully explored through the CCK8 experiment (Figure 6A, Supplementary Figure S3). the PDGFRA expression levels in IHH4 and 8505C cell lines were much higher than that of normal thyroid cells and other DTC cells (Figure 6B). Therefore, the compounds that exert its anticancer activity in a PDGFRA-dependent manner have better effect on IHH4 and 8505C cell lines than the others. Then Oxyberberine, 4',5,7-trimethoxyflavone, and Glycyrol were selected according to this screening principle. Radioiodine uptake experiments showed that among the three compounds, 4’,5,7-trimethoxyflavone had the best enhancement on the uptake of radioiodine by IHH4 cells (Figure 6C, Supplementary Figure S4). Later, this study mainly explored the mechanism of 4’,5,7-trimethoxy-flavone enhancing radioiodine uptake capacity of thyroid cancer. Western blot assay showed that 4’,5,7-trimethoxyflavone inhibited the phosphorylation activation of PDGFRA and p38 MAPK in a concentration- and time-dependent manner (Figures 6D,E). The CETSA was further performed to detect the binding of 4’,5,7-trimethoxyflavone to PDGFRA. The results showed that 4’,5,7-trimethoxyflavone increased the thermal stability of PDGFRA, indicating that the compound could bind to PDGFRA (Figure 6F). This result was consistent with the prediction of molecular docking. Only when NIS is accurately located on the membrane, it can play its role in radioiodine uptake. We further showed that 4’,5,7-trimethoxyflavone could increase NIS expression in cell membrane and cytoplasm at the same time (Figure 6G).

## 4 Discussion and Conclusion

Thyroid cancer is one of the most common endocrine tumors with an increasing incidence worldwide, among which RAIR-TC has a low survival rate due to its low radioiodine uptake rate. PDGFRA was found to reduce the expression of TG and NIS by disrupting the transcriptional activity and nuclear localization of TTF1, thus affecting the uptake of radioiodine (Lopez-Campistrous et al., 2016). Through the analysis of public database, this study also found that PDGFRA was closely connected with the genes and pathways related to radioiodine absorption in thyroid cancer (Figures 2B,C). In addition, PDGFRA also has an impact on the prognosis of thyroid cancer (Figure 2D), which is consistent with reported research (Lin et al., 2021). Currently, Sorafenib is the only PDGFRA inhibitor clinically used for the treatment of RAIR-TC in China, but Sorafenib can’t enhance the overall survival of patients (Brose et al., 2014). Therefore, further development of novel inhibitors of PDGFRA is still needed.

To build a PDGFRA inhibitors screening model with high accuracy, we combined the random forest model, SVM based prediction model, and molecular docking model. The Random forests model is commonly used for the predictive performance of multiple important drug transporters, targets, and drug properties, and is currently used to measure the importance of feature descriptors (Svetnik et al., 2003; Xu and Li, 2018). The Random Forest model, which takes the classification accuracy as the criterion function, has the advantages of high accuracy and good robustness. Screening the most important feature descriptors with Random Forest model first can greatly increase the accuracy of subsequent screening of PDGFRA inhibitors. SVM solves the classification problem by mapping data to a higher-dimensional space using nonlinear kernel functions to find the optimal separation hyperplane. Although the SVM has difficulties in multi-classification and large-scale training samples, it has good robustness and strong generalization ability for the classification of small sample data. The SVM based prediction model has been widely used in the field of chemical informatics to discover and design new drugs with excellent biological activities (Yabuuchi et al., 2011). Based on the SVM based prediction model and Random Forest model, this study constructed a screening model for PDGFRA inhibitors with 94% accuracy, and the prediction accuracy for positive compounds even reached 97%, confirming the reliability of the subsequent screening of 30 potential PDGFRA inhibitors. To further improve the accuracy of drug screening and reduce experimental costs, a molecular docking model was constructed to investigate the binding of candidate drugs to the active region of PDGFRA. Molecular Docking is mainly based on the principles of geometric matching and energy matching, using computer algorithms to predict the best binding mode for the receptor-ligand complex (Kitchen et al., 2004). Based on the docking results, 19 candidate compounds were further screened to bind PDGFRA active region.

According to the optimal 10 candidate compounds obtained by molecular docking results, the CCK8 experiments were performed to determine their inhibitory effects on different thyroid cancer cells. Since the expression level of PDGFRA in IHH4 and 8505C cell line was higher than that in other DTC cell lines, PDGFRA inhibitors should have better inhibitory effects on IHH4 and 8505C cell viability than other DTC cell lines (TPC1, BCPAP) and normal thyroid cells (Nthy-ori-3–1). Therefore, Oxyberberine, 4’,5,7-trimethoxyflavone, and Glycyrol that have better anticancer activity on IHH4 and 8505C were selected for further experimental verification (Figure 6A, Supplementary Figure S3). Radioiodine uptake experiments showed that among the three compounds, 4’,5,7-trimethoxyflavone had the best enhancement on the uptake of radioiodine by IHH4 cells (Supplementary Figure S4). Though the enhancement on the radioiodine uptake of 4’,5, 7-trimethoxyflavone in IHH4 cells was still not up to the uptake of radioiodine by normal thyroid cells (Nthy-ori-3–1), but it had a better effect on improving the uptake of radioiodine in RAIR-TC. Unfortunately, this study didn’t compare whether the combination of 4’,5,7-Trimethoxyflavon and radioactive iodine improved the therapeutic effect of radioactive iodine, and relevant studies will be carried out in the future.

In thyroid cancer, the MAPK signaling pathway is closely related to radioiodine uptake capacity, and its abnormal activation will lead to the loss of the expression of genes required for thyroid hormone biosynthesis, including NIS, TPO, and TG, thus reducing the radioiodine uptake capacity of thyroid cancer (Nagarajah et al., 2016). MAPK pathway is also regulated by PDGFRA (Rosenkranz et al., 2000; Hayashi et al., 2015). Therefore, we hypothesized that 4’,5,7-trimethoxyflavone promoted NIS expression or membrane localization by inhibiting PDGFRA-MAPK pathway, thus improving radioiodine uptake in thyroid cancer. This hypothesis was confirmed by western blot assays *in vitro*, 4’,5,7-trimethoxyflavone inhibited PDGFRA phosphorylation and the activation of the downstream MAPK signaling pathway, mainly the p38 MAPK pathway, and thus restored NIS expression in both membrane and cytoplasm (Figures 6D,E,G). Moreover, 4’,5,7-trimethoxyflavone has been confirmed as an inhibitor of PDGFRA through CETSA and western blot assays (Figure 6F).

4’,5,7-trimethoxyflavone has been observed to show several bioactivities, such as anti-allergy (Kobayashi et al., 2015), anti-Alzheimer (Youn et al., 2016), anti-cancer (Zheng et al., 2010), anti-inflammatory (During and Larondelle, 2013), and vasorelaxation effect (Tep-areena and Sawasdee, 2010), as well as enhancing the uptake of radioiodine in RAIR-TC as current study indicated. Metabolism of 4’,5,7-trimethoxyflavone mainly contains demthylation and phase II conjugation (Mekjaruskul et al., 2012), and pharmacokinetics of 4’,5,7-trimethoxyflavone showed dose- and time-dependence (Elhennawy and Lin, 2018). The difference in 4’,5,7-trimethoxyflavone dose influenced the plasma concentration-time curve of single intravenous administration, the mean residence time, the dose normalized maximum plasma concentration (C_max_) and area under the plasma concentration-time curve of single oral administration. Oral administration of 4’,5,7-trimethoxyflavone for 1 week could accelerate elimination and reduce plasma exposure. Despite the pharmacokinetic defects of 4’,5,7-trimethoxyflavone, it is still a promising drug lead with wide application.

In conclusion, with the successful construction of PDGFRA inhibitor screening model using machine learning and molecular docking approaches, we identified novel active ingredients from TCM that could improve the iodine sensitivity of RAIR-DTC. Among the selected TCM ingredients, 4’,5,7-trimethoxyflavone showed the best *in vitro* activity, which significantly upregulated the expression of NIS by targeting PDGFRA and inhibited PDGFRA activation, and therefore could enhance the radioiodine uptake capacity of thyroid cancer cells. This study provides support for the development of better RAIR-DTC therapy and potentially promotes the application of TCM in thyroid cancer treatment.

## Data Availability

The original contributions presented in the study are included in the article/Supplementary Material, further inquiries can be directed to the corresponding authors.
