# Supplementary material for: In Silico Screening and Validation of PDGFRA Inhibitors Enhancing Radioiodine Sensitivity in Thyroid Cancer
Source: Front Pharmacol. 2022 May 12;13:883581. doi: 10.3389/fphar.2022.883581 (PMC9133930; doi:10.3389/fphar.2022.883581)
Supplement: Supplementary file 3 [file DataSheet1.DOCX]

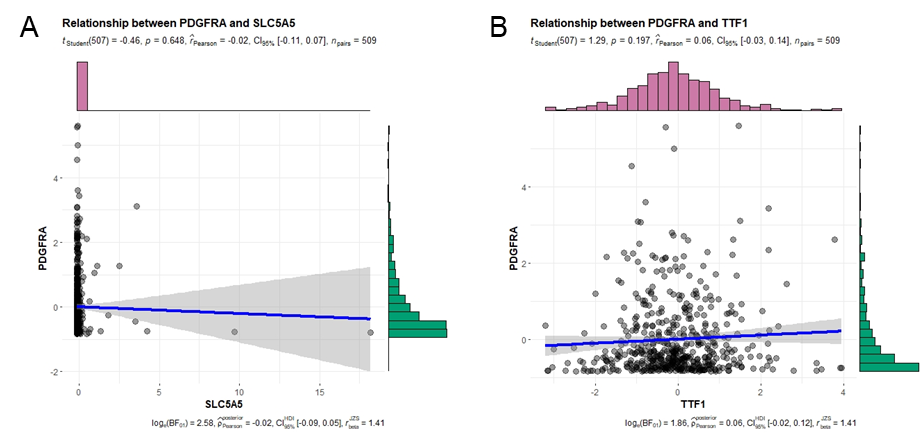


Supplementary figure 1. Correlation between PDGFRA and SLC5A5 (A), TTF1 (B) based on TCGA database.


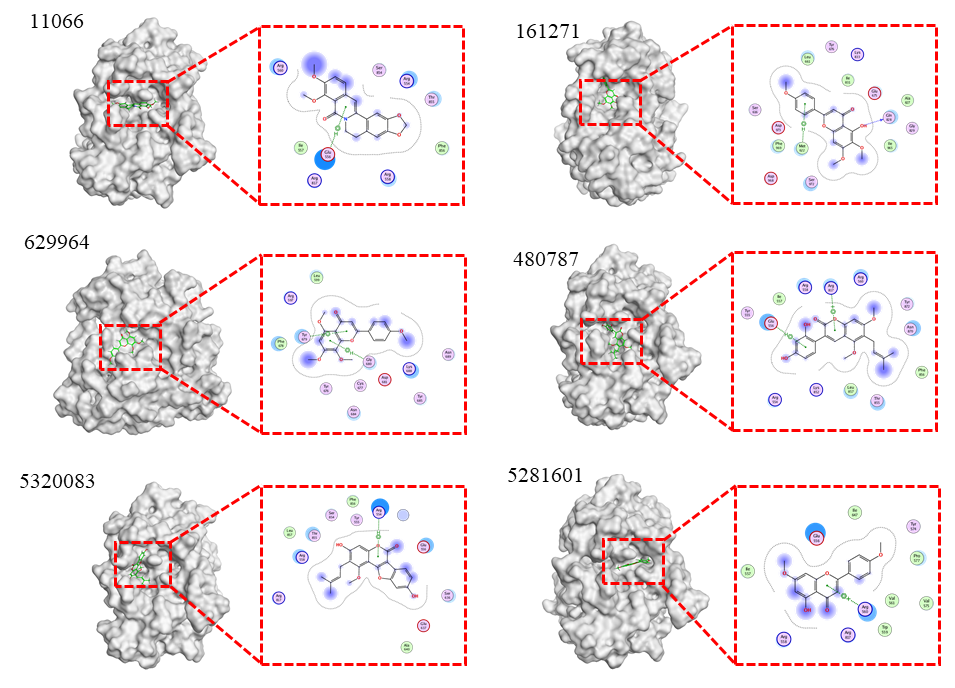


Supplementary figure 2. Docking interaction of the top compounds with PDGFRA. The serial numbers are PubChem CID of ligands. Protein of PDGFRA showed as white surface model, the ligand is showed as green stick model. Hydrogen bond is showed as dotted line. In 2D interaction diagram, ligand is showed as Chemical formula, residues are shown in purple circles marked with their names. Hydrogen bond is showed as green dotted line.


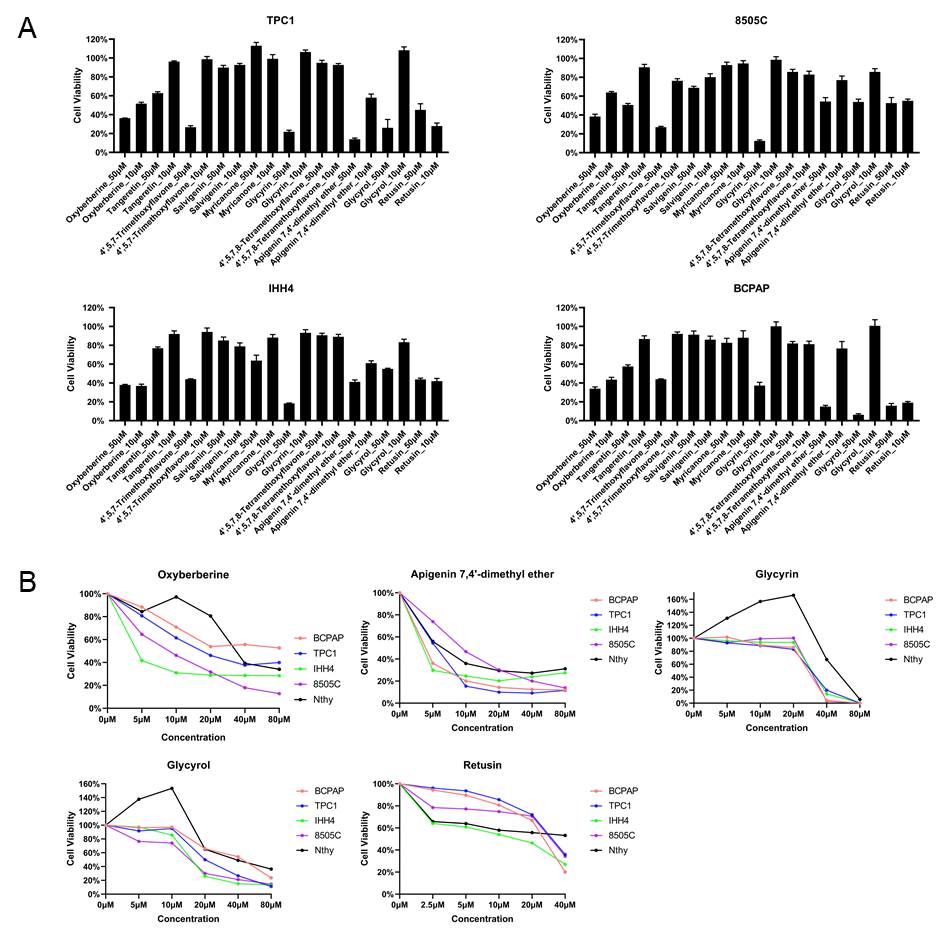


Supplementary figure 3. The effects of ten potential PDGFRA inhibitors on the viability of thyroid cancer cell lines through the CCK8 experiment. (A) Preliminary experimental results of the effects of 10 potential PDGFRA inhibitors (10μM, 50μM) on the cell activity of TPC1, BCPAP, IHH4, 8505C cell lines. Oxyberberine, Apigenin 7,4 '-dimethyl ether, Glycyrin, 4’,5,7-trimethoxyflavone, Glycyrol, and Retusin with good inhibitory effect on cell viability were selected by preliminary experimental results. (B) The effect of Oxyberberine, Apigenin 7,4’-dimethyl ether, Glycyrin, Glycyrol and Retusin on the viability of thyroid carcinoma cells and normal thyroid cells.


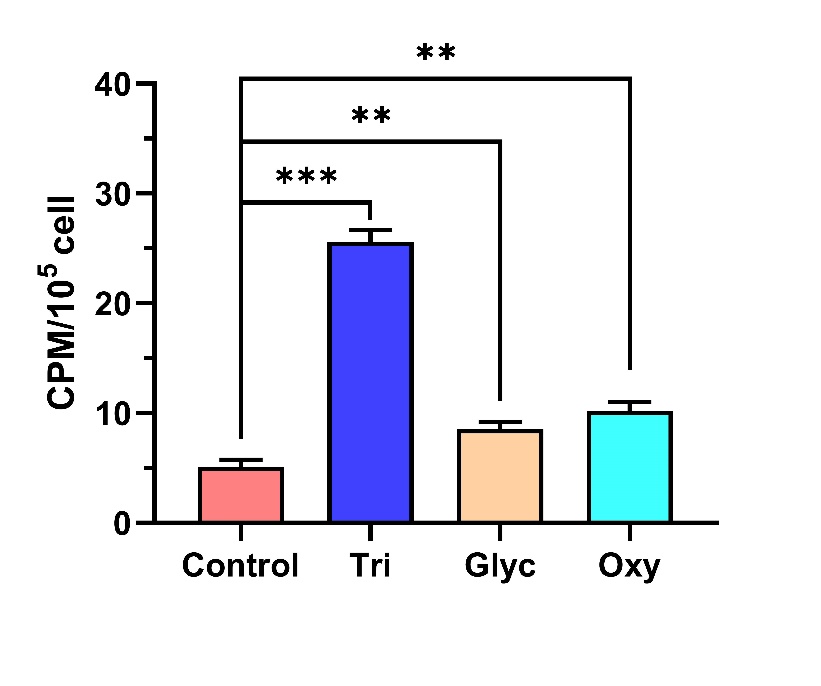


Supplementary figure 4. The effect of 4',5,7-trimethoxyflavone, Glycyrol and Oxyberberine on radioiodine uptake capacity of IHH4 cell line. ***, p<0.001; **, p<0.01, compared with control group. Tri: 4',5,7-trimethoxyflavone; Glyc: Glycyrol; Oxy: Oxyberberine.
